# Guest Editorial: Responding to Blood Lead Levels < 10 μg/dL

**DOI:** 10.1289/ehp.10703

**Published:** 2008-02

**Authors:** Mary Jean Brown, George G. Rhoads

**Affiliations:** Chief, Lead Poisoning Prevention Branch, Centers for Disease Control and Prevention, Atlanta, Georgia, E-mail: mjb5@cdc.gov; Chair, Advisory Committee on Childhood Lead Poisoning Prevention, Associate Dean, School of Public Health, University of Medicine and Dentistry of New Jersey, Piscataway, New Jersey

Miranda et al. (2007) in the August issue and Jusko et al. (2008) in the present issue of *Environmental Health Perspectives* provide additional evidence of adverse health effects in children at blood lead levels (BLLs) < 10 μg/dL—the Centers for Disease Control and Prevention’s (CDC) BLL of concern. A surprising feature of the new data in children with BLLs < 10 μg/dL is the steepness of the blood lead–IQ curve at these low levels. Nonlinear modeling conducted by Canfield et al. (2003) suggested a 7.4-point IQ effect (roughly one-half standard deviation) as lifetime average BLL went from 1 to 10 μg/dL—more than twice as strong a relationship as shown by most estimates at higher exposures (Treatment of Lead-exposed Children Trial Group 1998). It may reasonably be asked whether such a strong relationship is plausible, particularly as there are no directly relevant animal or *in vitro* studies that demonstrate a steeper slope for adverse effects of lead exposure at lower BLLs than observed at higher levels.

The CDC’s National Health and Nutrition Examination Survey (NHANES) data indicate that the entire population of U.S. children 1–5 years of age enjoyed a decline in geometric mean BLLs from about 15 μg/dL in the late 1970s to < 2 μg/dL in 2002 (CDC 2005a), but there is no agreement that IQs have increased by 7 points. Reading scores for 9-year-old children in the United States, tracked by the National Center for Education Statistics, show little change from 1980 to 1999, a period that would have corresponded to the years of greatest blood lead decline in the toddler years of the tested children (Perie and Moran 2005). Thus, the interpretation of the recent results may not be obvious. New studies using designs that exclude or control potential confounding by pica, which can indicate an underlying developmental delay, or by environmental hygiene, indicating more widespread lead contamination, would be especially helpful in assessing these low-level effects.

In the past, the CDC has responded to reports of adverse health effects at BLLs below the level previously thought to cause harm, by lowering the BLL that defines a child as lead poisoned. However, in 2005, the CDC and its Advisory Committee on Childhood Lead Poisoning Prevention, after a review of the available evidence, determined that children with BLLs < 10 μg/dL should not be considered lead poisoned as the term is used in the clinical setting (CDC 2005b). In addition, the CDC found that *a*) no effective, feasible interventions to reduce BLLs in this range have been demonstrated; *b*) no threshold for adverse effects has been identified; and *c*) given current laboratory methods, risk for misclassification of children is high. Thus, the approach of arbitrarily defining a new, lower BLL of concern was rejected. This decision also is consistent with the recently released recommendations of the U.S. Preventive Services Task Force (2006) “against routine screening for elevated blood lead levels in asymptomatic children aged 1 to 5 years who are at average risk.”

Rather, the CDC recommends a multitiered approach that includes case management of children with BLLs > 10 μg/dL coupled with an increased focus on primary prevention through the control and elimination of lead in children’s environments. The elements of this strategy include:

- State-based strategic plans to eliminate childhood lead poisoning through a systematic society-wide effort that includes legislative and enforcement efforts to control lead paint hazards, particularly in the highest-risk housing (CDC 2007)
- A partnership between the CDC, the Environmental Protection Agency, and the U.S. Department of Housing and Urban Development to enforce the Residential Lead-Based Paint Hazard Reduction Act (1992) particularly in housing where children have repeatedly been identified as having elevated BLLs
- Elimination of lead in consumer products that are marketed to children
- Recommendations that regulatory agencies abandon the practice of using a BLL of 10 μg/dL as the threshold for enforcement activities.

These strategies focus on primary prevention of lead exposure to children, an approach that is in agreement with the conclusions of Jusko and colleagues (2008) as expressed in their earlier report from the same cohort (Canfield et al. 2003).

In 1990, the nation adopted an ambitious goal to eliminate BLLs > 10 μg/dL as a public health problem by 2010. Recent data indicate that this goal is in sight. However, studies such as those by Miranda et al. (2007) and Jusko et al. (2008) sound a cautionary note. For the foreseeable future, lead will continue to contaminate the environment. Therefore, even after the 2010 goal is achieved, primary prevention efforts must be maintained to ensure that lead sources in children’s environments are controlled or eliminated before children are exposed and that surveillance systems are in place to ensure that these efforts are effective.

Therefore, even after the 2010 goal is achieved, primary prevention efforts must be maintained to ensure that lead sources in children’s environments are controlled or eliminated before children are exposed and that surveillance systems are in place to ensure that these efforts are effective.

## Figures and Tables

**Figure:**
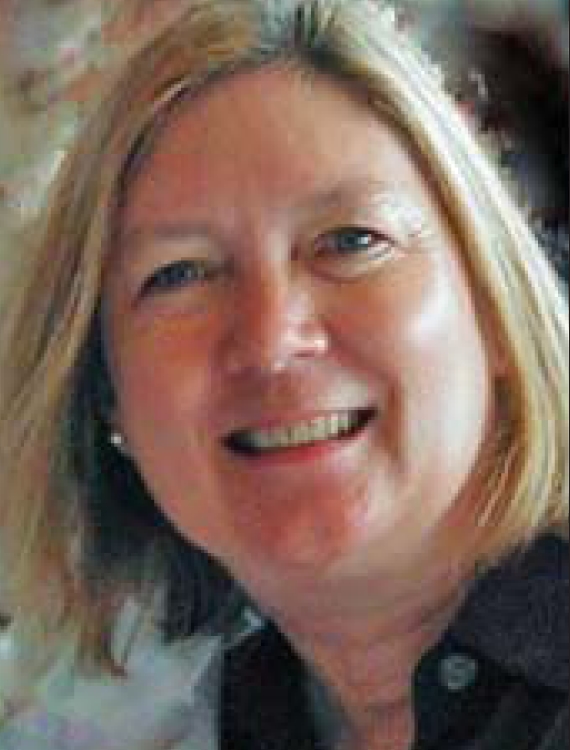
Mary Jean Brown

**Figure:**
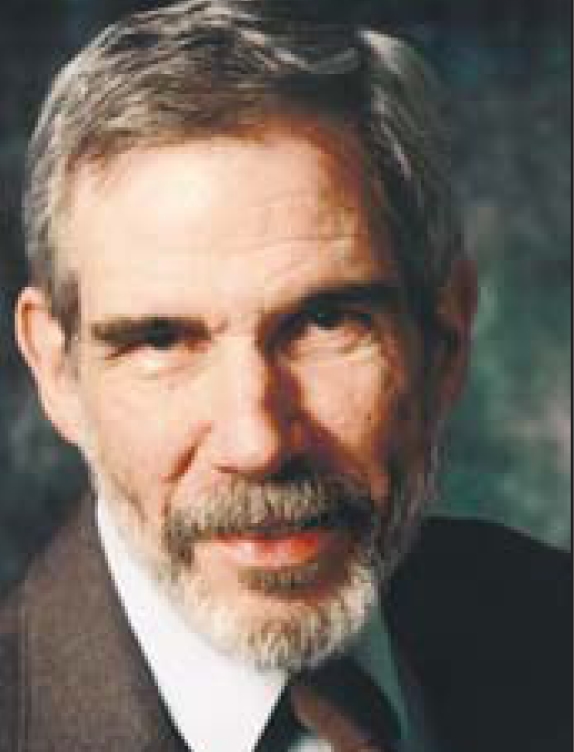
George G. Rhoads
